# Can Nocebo Effects Be Reduced via Open‐ and Closed‐Label Counterconditioning?

**DOI:** 10.1002/ejp.70248

**Published:** 2026-03-12

**Authors:** Simone Meijer, Andrea W. M. Evers, Kaya J. Peerdeman, Henriët van Middendorp

**Affiliations:** ^1^ Health, Medical and Neuropsychology Unit Leiden University Leiden the Netherlands; ^2^ Center for Interdisciplinary Placebo Studies Leiden (IPS) Leiden the Netherlands; ^3^ Leiden Institute for Brain and Cognition (LIBC) Leiden the Netherlands; ^4^ Medical Delta, Erasmus University Rotterdam, Leiden University & Delft University of Technology Rotterdam, Leiden, Delft the Netherlands

**Keywords:** counterconditioning, extinction, nocebo effect, nocebo hyperalgesia, open‐label, pressure pain

## Abstract

**Background:**

The overwhelming evidence of nocebo effects adversely affecting the experience of physical symptoms, such as pain, puts emphasis on the study of possible strategies to reduce nocebo effects. Counterconditioning, during which a previously conditioned effect is reversed, has been shown to be a possible promising strategy in a previous study in an open‐label design, in which people were informed about the use of inert treatments and the counterconditioning procedure. However, it is unclear whether open‐label counterconditioning is as effective as closed‐label procedures in comparison to extinction.

**Methods:**

In a randomised controlled trial, we investigated in 66 healthy female participants whether conditioned nocebo effects on pressure pain can be reduced via open‐label counterconditioning, closed‐label counterconditioning, or extinction.

**Results:**

A significant reduction of nocebo effects was found after open‐label counterconditioning (*d* = 1.13), closed‐label counterconditioning (*d* = 0.69), and extinction (*d* = 0.66). Open‐label counterconditioning was more effective than extinction (*d* = 0.85), reversed the nocebo effect, and also resulted in a placebo effect. Closed‐label counterconditioning was more effective than extinction (*d* = 0.45) in the sensitivity analyses of nocebo responders only. Finally, larger placebo expectancies predicted a higher response to the open‐ and closed‐label counterconditioning procedures, but not extinction.

**Conclusions:**

These results show that particularly open‐label counterconditioning is an effective method to modulate nocebo effects on pressure pain. This provides promise in designing non‐deceptive learning‐based treatments to reduce nocebo effects in diverse patient groups, including chronic pain disorders.

**Significance Statement:**

Few studies have investigated the efficacy open‐label counterconditioning to reduce nocebo effects or have compared it to deceptive reduction strategies. The current study demonstrates that open‐label counterconditioning is as effective as more deceptive nocebo‐reducing strategies and may thus be a promising new method for reducing nocebo effects in a non‐deceptive and ethical manner. Results can be helpful while designing learning‐based treatments to reduce nocebo effects in patients with, for example, chronic pain disorders.

**Trial Registration:**

ClinicalTrials.gov identifier: NCT05284383

## 1 Introduction

Nocebo effects (i.e., adverse treatment outcomes not attributable to active treatments) can negatively influence physical symptoms and can be induced via learning mechanisms as classical conditioning and verbal suggestions (Benedetti et al. 2007; Colloca et al. 2008; Petersen et al. 2014; Thomaidou et al. 2020). Research indicated conditioned nocebo effects can be reduced by counterconditioning, during which the original unconditioned stimulus (US) (e.g., pain increase) that was paired to a neutral stimulus (e.g., activation of a sham device), is replaced by another US of opposite valence (e.g., pain decrease) (Bartels et al. 2017; Meijer et al. 2023; Thomaidou et al. 2020). Studies on fear conditioning have shown that counterconditioning effectively reduces conditioned effects, but results on superiority of counterconditioning over extinction (during which the CS and US not paired, leading to people learning that the US and CS are no longer associated) are mixed (Jozefowiez et al. 2020). Results in nocebo research indicated that counterconditioning is more effective than extinction; hence, counterconditioning could be especially promising for reducing nocebo effects in both experimental settings and clinical practice (Bartels et al. 2017; Meijer et al. 2023; Thomaidou et al. 2020).

Experimental research typically used deceptive (counter)conditioning paradigms where participants are not informed about US and CS manipulation (Bartels et al. 2014, 2017; Colagiuri et al. 2015; Colloca et al. 2008, 2010; Thomaidou et al. 2020). In clinical practice, deceptive procedures are not appropriate, because deception could harm trust in the healthcare provider and treatment (Miller et al. 2005; Peerdeman et al. 2021). Open‐label counterconditioning, in which people are informed about the use of inert treatments and the counterconditioning procedure, provides a non‐deceptive opportunity for reducing nocebo effects. Although open‐label placebo procedures have been demonstrated to be effective (Carvalho et al. 2016; Kaptchuk et al. 2010; Kelley et al. 2012; Kleine‐Borgmann et al. 2019; Locher et al. 2017; Meeuwis et al. 2019; Schaefer et al. 2016, 2018a), only one study investigated open‐label counterconditioning on pain, showing that open‐label counterconditioning could reduce nocebo effects induced by open‐label conditioning (Meijer et al. 2023). However, in daily life nocebo effects are typically unconsciously conditioned. Additionally, it is unknown whether open‐label counterconditioning is as effective as closed‐label counterconditioning and how it compares to closed‐label extinction, which would be comparable to a natural history group, as no active intervention is provided.

The current study aimed to investigate reduction of conditioned nocebo effects on pressure pain through open‐ or closed‐label counterconditioning (combined with suggestions) or extinction. To this aim, we first tested whether a nocebo response was established after closed‐label nocebo conditioning and suggestions in a healthy sample. Secondly, we compared nocebo reduction upon each procedure. We hypothesized that: (1) participants rate conditioned trials as significantly more painful than control trials upon nocebo conditioning, indicating a nocebo effect; (2) all three reduction methods reduce the nocebo effect; and (3) the reduction strategies differ in efficacy (primary analysis), with both counterconditioning methods yielding a larger reduction than extinction; (4) open‐ and closed‐label counterconditioning are equivalently efficacious; and (5) both open‐ and closed‐label counterconditioning, but not extinction, reverse the nocebo effect into a placebo effect.

## 2 Method

### 2.1 Ethics Statement

This study was approved by the Psychology Research Ethics Committee of Leiden University (reference number CEP18‐1114/442) and pre‐registered on ClinicalTrials.gov. All participants gave written informed consent and were reimbursed by €15 in cash or study credits. Participants could not be fully informed about the study procedure at the time of informed consent due to the nature of the study but were fully debriefed upon the end of their study visit.

### 2.2 Participants

The sample size required was calculated using G*Power 3.1. Besides the primary analysis (initial comparison of the three reduction methods), we wanted to accurately examine the pairwise comparisons of each counterconditioning method with extinction (hypothesis 3). The sample size was therefore calculated for a 3 × 2 mixed‐model ANOVA (two‐sided, alpha = 0.05, desired power 0.80) and the expected effect size was *d* = 0.86, based on two studies that compared counterconditioning with extinction for reducing nocebo effects on pain (Meijer et al. 2023; Thomaidou et al. 2020). According to the sample size calculation, 23 participants were needed per group. Since the design consisted of 3 groups in the second phase, we aimed for a total of 69 participants.

Participants were recruited through flyers at Leiden University and online via Facebook, as well as via the University's online recruitment system Sona (Sona systems, Tallin, Estonia). All participants had to be female, between 18 and 35 years old, and to have a good understanding of written and spoken Dutch or English. The counterconditioning procedure tested in the current study, once found to be effective in healthy participants, is intended to be used in future research with patients with chronic pain, such as fibromyalgia (Meijer et al. 2022). As fibromyalgia is more prevalent in women (Marques et al. 2017), only female participants were tested in the current study to avoid the possible influence of gender differences.

Exclusion criteria were severe somatic or psychiatric morbidity (e.g., heart/lung diseases, DSM‐5 psychiatric disorders), Raynaud's disease, chronic pain complaints at present or in the past (≥ 3 months), current pain complaints (≥ 2/10 on Numeric Rating Scale (NRS)), current use of medication, injuries on the non‐dominant hand, refusal/inability to remove nail polish or artificial nails on the thumbnail of the non‐dominant hand for the experiment, colour blindness, and pregnancy or breastfeeding. Participants were excluded from further participation if their sensory discrimination was poor, that is, if they were unable to distinguish between three different pressure intensities, or if a pain intensity of 5/10 on NRS was not reached at maximum pressure levels. Participants were asked not to consume alcohol, recreational drugs, painkillers, and/or sleep medication in the 24 h prior to testing.

### 2.3 Design

**FIGURE 1:**
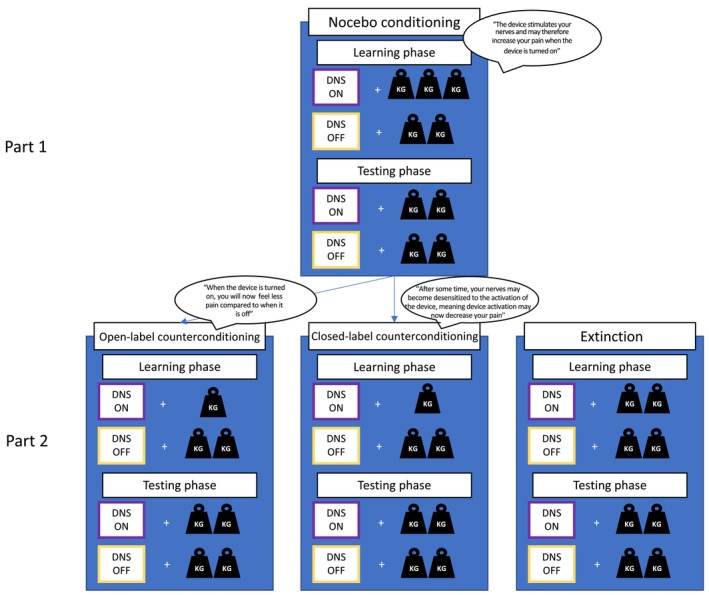
Overview of the study design. In part 1 (nocebo induction), participants underwent a nocebo conditioning procedure. During the learning phase of nocebo conditioning, participants received moderate pain (5–6 on 0–10 Numeric Rating Scale [NRS]) during ‘DNS on’ trials and slight pain (2.5–3.5 on 0–10 NRS) during ‘DNS off’ trials. In the test phase participants received slight pain stimuli for all trials. In part 2 participants were randomly assigned (1:1:1) to one of three groups: open‐label counterconditioning, closed‐label counterconditioning or extinction. During both forms of counterconditioning, ‘DNS on’ trials were now paired with minimal pain (0–1 on 0–10 NRS) and ‘DNS off’ trials with slight pain in the learning phase. These groups only differed in verbal suggestions provided. During extinction, all trials were paired with a slight pain intensity. The test phases in all groups in part 2 were identical to the test phases in part 1. DNS, Dermal Nerve Stimulation.

A randomised controlled trial with a between‐within subjects design was employed, consisting of two parts (Figure 1). Participants were randomly assigned to one of three groups (1:1:1). A randomization list was made by an independent person and group allocation was noted down on paper and inserted into an opaque envelope, which was opened after the pressure pain calibration procedure to prevent experimenter bias during calibration. In part 1 (nocebo induction), all participants underwent a closed‐label nocebo conditioning procedure, regardless of group. In part 2, participants underwent one of three nocebo‐reduction strategies: open‐label counterconditioning, closed‐label counterconditioning, or closed‐label extinction. Since most experimental manipulations contained verbal instructions and self‐report measures were used, neither the experimenter nor the participant could be fully blinded to group allocation.

### 2.4 Pain Induction

To induce pain, pressure‐pain stimuli were applied to the thumbnail of the non‐dominant hand using a custom‐made automated pneumatic stimulator, made by SOLO (Support for Research, Laboratories and Education, Leiden University). The design of the pneumatic stimulator is based on a pressure device used in several previous studies (Jensen et al. 2009; Meijer et al. 2023; Petzke et al. 2003). The handpiece of the stimulator has a plastic piston that applies pressure via a 1 cm^2^ hard rubber probe. The handpiece has a cylinder opening where participants can insert their thumb, placed such that the probe contacts the middle of the thumbnail. The thumbnail was selected as a neutral location to repeatedly and safely deliver pressure stimuli, as has been previously used and reported on for both healthy and clinical samples (Jensen et al. 2009). Pressure stimulus duration was set at 2.5 s, with an inter‐stimulus interval of 30 s. The minimum intensity of pressure given was set at 0.5 kg/cm^2^, while the maximum was set at 13 kg/cm^2^, which has previously been established to be a safe amount of pressure on the thumbnail (Lacourt et al. 2012). Pressure pain was chosen, as it closely taps into the specific sensitivity to pressure and mimics the real‐life experience of patients with musculoskeletal disorders (Petzke et al. 2003; Wolfe et al. 1990), and procedures of the current study were intended to be used in future studies with such patients (Meijer et al. 2022).

### 2.5 Pressure Pain Calibration

A calibration procedure was conducted in order to find the optimal pressure intensity for minimal pain (0–1/10 NRS), slight pain (2.5–3.5/10 NRS), and moderate pain (5–6/10 NRS) for the individual participant, to be used in parts 1 and 2 of the experiment. The calibration procedure consisted of three parts (ascending series, random series, calibration check) and was identical to the procedure described in a design paper on a counterconditioning treatment protocol (Meijer et al. 2022).

### 2.6 Experimental Procedures

#### 2.6.1 Sham Device

During the experiment, a sham Transcutaneous Electrical Nerve Stimulation (TENS) device combined with a message indicating its (de)activation on a screen was used as a conditioning stimulus. To avoid potential interference by participants' possible previous experiences or knowledge on the functions of TENS, the device was referred to as a Dermal Nerve Stimulation (DNS) device. Two electrodes were attached below each other on the radial side of the participants' non‐dominant forearm. Participants were told that the DNS device can stimulate nerve fibres, which may lead to people becoming more or less sensitive to incoming stimuli, such as pain. They were also given an information leaflet (Appendix S1) which further explains the function of the DNS device. Right before the start of both nocebo induction and reduction (except for extinction), more specific verbal suggestions regarding the function of the device were provided, depending on group (see Sections 2.6.2 and 2.6.3).

The messages indicating (de)activation of the device were presented to participants on a computer screen, in purple or yellow text (colours associated with either activation or deactivation were counterbalanced across participants). The messages were displayed for 3.5 s, starting 1 s before the pressure was administered. Participants were instructed to keep paying attention to the screen. In between stimuli, a fixation cross was shown.

#### 2.6.2 Nocebo Conditioning (Part 1)

The nocebo conditioning part of the study consisted of a learning and testing phase (see Figure 1). In the learning phase, a button‐press on the sham DNS device by the experimenter combined with a computer screen message indicating the activation of the DNS device (“DNS ON”), was repeatedly paired with a moderate‐intensity pressure pain stimulus (pressure scored as 5–6 on 0–10 NRS for that participant). Another computer screen message indicating the deactivation of the sham DNS device (“DNS OFF”) was repeatedly paired with a slight‐intensity pressure pain stimulus (2.5–3.5 on 0–10 NRS). In total, the learning phase consisted of 10 experimental trials (“DNS ON trials”) and 10 control trials (“DNS OFF trials”), presented in a standard pseudorandom order (maximally 2 stimuli of the same trial type (experimental or control) could follow each other). The testing phase consisted of 3 experimental and 3 control trials in pseudorandom order (maximally 2 stimuli of the same trial type (experimental or control) could follow each other), all associated with a slight pressure pain intensity. Participants were given verbal suggestions about the DNS device before the start of conditioning and were told that the DNS device can stimulate the nerve fibres, leading to a heightened sensitivity to incoming stimuli such as pain. They were instructed that during part 1, whenever the DNS device was turned on, they may feel more pain compared to when the DNS device was turned off.

#### 2.6.3 Nocebo Reduction (Part 2)

For all groups, the learning phase of the nocebo‐reduction part consisted of 10 experimental and 10 control trials, and the testing phase was identical to the testing phase for the nocebo‐induction part.

The open‐label counterconditioning procedure was identical to the closed‐label procedure, apart from the verbal suggestions given to participants. They were told that the DNS device was in fact sham and therefore inactive and that their pain was manipulated by the experimenter in order to teach them a contingency between the device and pain increase. They were then instructed that now this contingency would be changed, by pairing the device to a decrease in pain.

The closed‐label counterconditioning procedure differed from the nocebo conditioning in part 1 such that a minimally painful pressure stimulus (0–1 on a 0–10 NRS) instead of a moderate‐intensity pressure pain stimulus now followed the “DNS ON” message. Participants were again given verbal suggestions about the DNS device and were now told that after a certain amount of time, the sensitivity‐increasing effects of DNS stimulation may wear off. They were then told that when administered for this long, DNS stimulation could decrease nerve sensitivity and thereby the incoming pain signal, meaning that during part 2, they may feel less pain when the DNS was turned on compared to when it was off.

In the extinction procedure, only slightly painful stimuli were given during all trials, in both the learning and the testing phase. No specific verbal suggestions regarding extinction were given; participants were merely told they would again receive a series of stimuli and that the screen would indicate whether the DNS device was turned on or off.

### 2.7 Self‐Report Ratings

A questionnaire including demographic and health questions was used to screen participants for inclusion. Furthermore, several validated questionnaires were used to measure baseline psychological characteristics: fear of pain was measured using the Fear of Pain Questionnaire III (FPQ‐III) (McNeil and Rainwater 1998), trait and state anxiety were measured using the State–Trait Anxiety Inventory, Trait Scale (STAI‐T) (Spielberger 1983) and State Scale Short Form (STAI‐Ss) (Marteau and Bekker 1992), locus of control was measured with the Multidimensional Health‐Related Locus of Control Scale (MHLOC) (Wallston et al. 1978). Locus of control refers to the individual's perception that an outcome is either the result of their own behaviour (internal locus of control) or that an outcome is in control of powerful others (subscale “Powerful Others”) or the result of fate, luck, or chance (subscale “Chance”), both indicating an external locus of control. Somatosensory amplification, or the tendency to experience a somatic sensation as intense, noxious, and/or disturbing, was measured using the Somatosensory Amplification Scale (SSAS) (Barsky et al. 1988). Finally, several dimensions of personality (i.e., psychoticism, neuroticism, extraversion, and social desirability) were measured using the Eysenck Personality Questionnaire Revised (Short Scale) (Eysenck and Eysenck 1984).

During the experiment, experienced pain intensity (“How painful would you rate the last stimulus on a scale of 0‐10?”) was reported after each pressure stimulus on a Numeric Rating Scale (NRS), ranging from 0 (no pain) to 10 (worst pain imaginable). Participants were allowed to use 1 decimal while scoring their pain. Additionally, expected pain (“How painful do you expect the upcoming stimulus to be on a scale of 0‐10?”) was reported right before every first and tenth experimental and first and tenth control trial of the learning phase and every first experimental and control trial of the testing phase. This was also reported on an NRS ranging from 0 (no pain) to 10 (worst pain imaginable).

An exit questionnaire consisted of questions on (1) what participants thought the aim of the experiment was (open‐ended); (2) level of focused attention during the experiment (0–10 NRS, with a higher score indicating more focused attention); (3) trustworthiness of the experimenter (on a scale of 0–10, with a higher score indicating more trustworthiness); (4) competence of the experimenter (on a scale of 0–10, with a higher score indicating more competence); and (5) whether participants adjusted pain ratings during the experiment to please the experimenter (on a scale of 0–10, with a higher score indicating a higher degree of adjusted answers and thus response bias).

Baseline and exit questionnaires were filled in using Qualtrics software (Qualtrics, Provo, Utah, United States). NRS scores were filled in by the participants on a computer using Eprime 3.0 software (Psychology Software Tools, Pittsburgh, PA).

### 2.8 Procedure

During the experimental procedure, the experimenter followed a standardised script to ensure procedures for each participant resembled each other closely. After the procedure was explained, participants signed the informed consent form. If participants were eligible based on the screening questions, participants completed all baseline questionnaires. Individual pressure pain levels were then calibrated. Next, part 1 of the experiment commenced (nocebo induction), followed by part 2 (nocebo reduction). Finally, participants completed the exit questionnaire and were debriefed and compensated for their participation. The experiment was conducted in a single session and took approximately 2 h, with 5‐min breaks in‐between the different parts of the calibration procedure and a 10‐min break between parts 1 and 2 of the experiment.

### 2.9 Statistical Analyses

All data were analysed using SPSS 25.0 (IBM SPSS Statistics, Chicago, Illinois, USA). Assumptions of all statistical tests were checked: normality was tested through examination of histograms and Shapiro–Wilk tests; for analyses of variance (ANOVAs), equality of variances was tested using Levene's tests; and boxplots were used to search for outliers. In case of violation, we used non‐parametric tests. The threshold of significance was set at *p* < 0.05, unless stated otherwise. One‐way ANOVAs were used to assess between‐group differences in calibration values, ability to focus during testing, trust in experimenter, perceived competence of the experimenter, and response bias.

To examine whether a significant nocebo effect was found after nocebo conditioning (hypothesis 1), a paired samples *t*‐test was performed, comparing the average NRS score of experimental trials with the average NRS score of control trials in testing phase 1. Additionally, pain scores on the first and last experimental and control trial of the learning phase were examined using a paired samples *t*‐test to check for sensitization. It was also tested (using a one‐way ANOVA) whether any group differences existed in the strength of the nocebo effect to check if this may influence results on group differences in the efficacy of the reduction strategies.

In the nocebo‐reduction part of the experiment, to determine whether the reduction of the nocebo effect within each group following nocebo induction was significant (hypothesis 2), three one‐sample *t*‐tests were performed. First, to calculate *nocebo reduction*, the nocebo effect in part 2 was subtracted from the nocebo effect in part 1. The *nocebo effect* was calculated by subtracting the average NRS score of control trials from the average NRS score of experimental trials in testing phase 1 and 2. In each group, the amount of reduction of the nocebo effect was compared to 0, with a significant positive deviation from 0 indicating a significant change of the nocebo effect. A Bonferroni correction was applied to correct for multiple testing and the threshold for significance was set at *p* < 0.016. Finally, to check for sensitization, pain scores on the 1st and last experimental and control trial of the learning phase were examined.

Then, to test our primary analysis (hypothesis 3) whether the three groups differed in amount of nocebo reduction, a 3 × 2 (group × time) mixed‐model ANOVA was conducted. We first examined the main effect of group on nocebo reduction. Three 2 × 2 (group × time) mixed‐model ANOVAs subsequently compared differences in nocebo reduction between all groups.

To test for equivalence of the open‐ and closed‐label counterconditioning procedures (hypothesis 4), the “two one‐sided tests” (TOST) approach was used (Lakens et al. 2018).

During the TOST, two one‐sided tests are performed, to test whether the difference between two conditions (in our study, the difference in nocebo reduction between both counterconditioning groups) is at least as large as the smallest effect size of interest. If not, the difference is too small to be (clinically) relevant and thus the conditions can be considered equivalent.

The upper and lower equivalence bound were based on the smallest effect size of interest, which was *η* ^2^ = 0.06 (a medium effect). Then the 90% confidence interval (CI) for the effect size of the difference between the reduction in the open‐label counterconditioning group and the closed‐label counterconditioning group was calculated using an SPSS syntax (retrieved from http://core.ecu.edu/psyc/wuenschk/SPSS/CI‐R2‐SPSS.zip) to determine whether the 90% CI fell within the previously established range of −0.06 to 0.06 (which would indicate equivalency).

Finally, in all groups separately a paired samples *t*‐test was performed to test whether the nocebo effect was reversed into a placebo effect after the reduction procedure (hypothesis 5). For this, the average NRS score of experimental trials was compared with the average NRS score after control trials in testing phase 2; a significant (negative) difference indicates a placebo effect.

Sensitivity analyses were conducted to assess the influence of excluding participants for whom no nocebo effect had been induced in phase 1 (i.e., participants for whom the difference between experimental vs. control trials in testing phase part 1 was zero or positive) from all analyses on nocebo reduction. For these participants, there was no nocebo effect to be reduced, which may lead to incorrect inferences on the effects of nocebo‐reduction strategies.

For the “expected nocebo effect” during both nocebo induction and reduction, three one‐way ANOVAs (six in total) compared the differences in expected nocebo effects between all groups, at the start of nocebo induction/reduction, halfway through nocebo induction/reduction, and at the start of the testing phase. Post hoc analyses were performed in case of any significant group differences to examine which groups differed from each other.

Additionally, it was explored using correlational analyses whether the nocebo effect and reduction of the nocebo effect were related to pain expectancies, that is, the difference between expected pain for the first ON and OFF trial of the testing phase of nocebo induction and reduction. A positive difference between ON and OFF trials would indicate participants would expect ON trials to be more painful; for nocebo induction this would indicate our manipulation was successful. For nocebo reduction, we would expect to find no difference (or a negative difference) between ON and OFF trials. As a manipulation check, six one‐way ANOVAs were conducted to test for group differences on the “expected pain difference” (i.e., difference in expected pain during ON and OFF trials) during different time points of nocebo induction and reduction.

For exploratory purposes, Pearson correlation coefficients were calculated between questionnaire scores for psychological characteristics with the nocebo effect (all participants combined) and nocebo reduction (for the subgroups separately). Sum scores of all questionnaires were calculated to use in the analyses.

Finally, the speed of reduction of the nocebo effect by both forms of counterconditioning and extinction was exploratively compared by examining the interaction between group (open‐label counterconditioning, closed‐label counterconditioning, and extinction) and time (10 nocebo scores—i.e., the difference between the experimental trials and control trials—throughout the learning phase of part 2) using a mixed ANOVA.

## 3 Results

### 3.1 Participants

Participants were recruited from March 2021 to March 2022. Out of 79 enrolled participants, 10 participants were excluded upon calibration because their pain threshold was too high (i.e., they did not reach a moderate pain level during calibration). Included participants were between 18 and 31 years old (*M* = 21.1, SD = 3.1).

**FIGURE 2:**
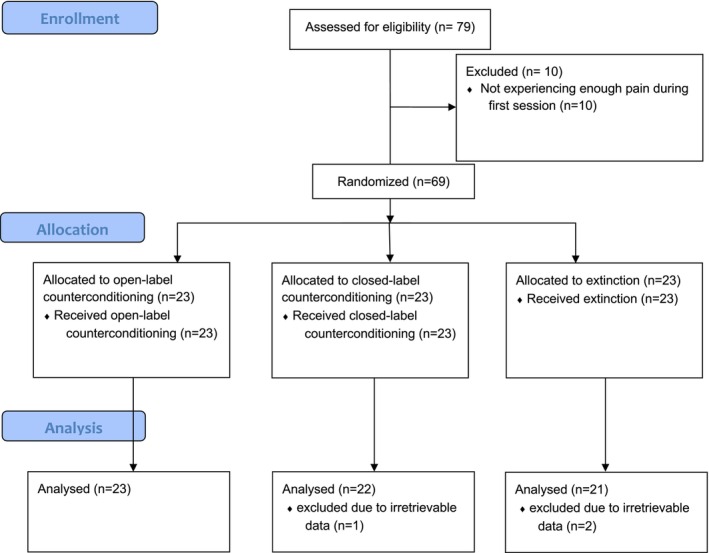
Flow diagram of the randomised controlled trial.

In total, 66 participants were included in the final analyses, because the data of 3 participants could not be retrieved due to an error in the saved files. A flow diagram is displayed in Figure 2. Descriptive data of calibration values and exit questionnaire scores are displayed in Appendix S2: Table B1. During screening, 5 people reported having current pain complaints (of lower than 2 on a 0–10 NRS, thus none were excluded), which were reported to be either muscle soreness from working out, mild menstrual pain, or a mild headache. No significant differences were found between groups for calibration values, trust in experimenter, perceived competence of the experimenter, and response bias.

### 3.2 Induction of the Nocebo Effect

The mean pain ratings on experimental and control trials during the testing phase of conditioning are displayed in Appendix S2: Figure B1 and Table 1. Experimental trials were rated as significantly more painful than control trials despite delivering the same painful stimuli in both types of trials; *t*(65) = 7.57, *p* < 0.001, *d* = 0.96, indicating a nocebo effect. These findings are in line with hypothesis 1. To check for sensitization, pain scores on the 1st and last experimental and control trial of the learning phase were examined. Participants did sensitise throughout nocebo conditioning, and they sensitised more on experimental trials (1.1 on NRS) compared to control trials (0.6 on NRS), meaning the difference between experimental and control trials became slightly larger throughout conditioning. Finally, no group differences were found in the strength of the nocebo effect, as no interaction between group and trial type (average score on ON vs. OFF trials) was found, *F*(2,63) = 0.329, *p* = 0.721.

**TABLE 1:** Group means and SDs for reported pain during learning and testing phases of nocebo induction and reduction, as well as the magnitude of the nocebo effect and the reduction of the nocebo effect.

	All participants		Subgroups		Closed‐label counterconditioning (*n* = 22)		Extinction (*n* = 21)	
	Nocebo conditioning (*n* = 66)		Open‐label counterconditioning (*n* = 23)					
	Mean	SD	Mean	SD	Mean	SD	Mean	SD
Nocebo Induction (part 1), learning phase								
NRS experimental trials	5.71	1.06	5.75	1.02	5.75	1.02	5.65	1.19
NRS control trials	3.39	1.05	3.38	0.98	3.50	1.11	3.32	1.09
Testing phase								
NRS experimental trials	4.33	1.32	4.22	1.44	4.22	1.18	4.54	1.35
NRS control trials	3.61	1.26	3.48	1.25	3.66	1.26	3.69	1.33
Nocebo effect	0.72	0.79	0.75	0.84	0.56	0.65	0.85	0.88
Nocebo reduction (part 2), learning phase								
NRS experimental trials			1.76	1.03	1.93	1.25	4.19	1.49
NRS control trials			4.23	1.16	4.42	1.44	4.10	1.31
Testing phase								
NRS experimental trials			3.25	1.12	3.74	1.67	4.41	1.79
NRS control trials			4.13	1.21	4.08	1.81	4.27	1.80
Nocebo effect			−0.88	0.93	−0.35	0.83	0.15	0.52
Reduction of nocebo effect (part 1–part 2)			1.63	1.44	0.93	1.36	0.60	0.91

### 3.3 Reduction of the Nocebo Effect

**FIGURE 3:**
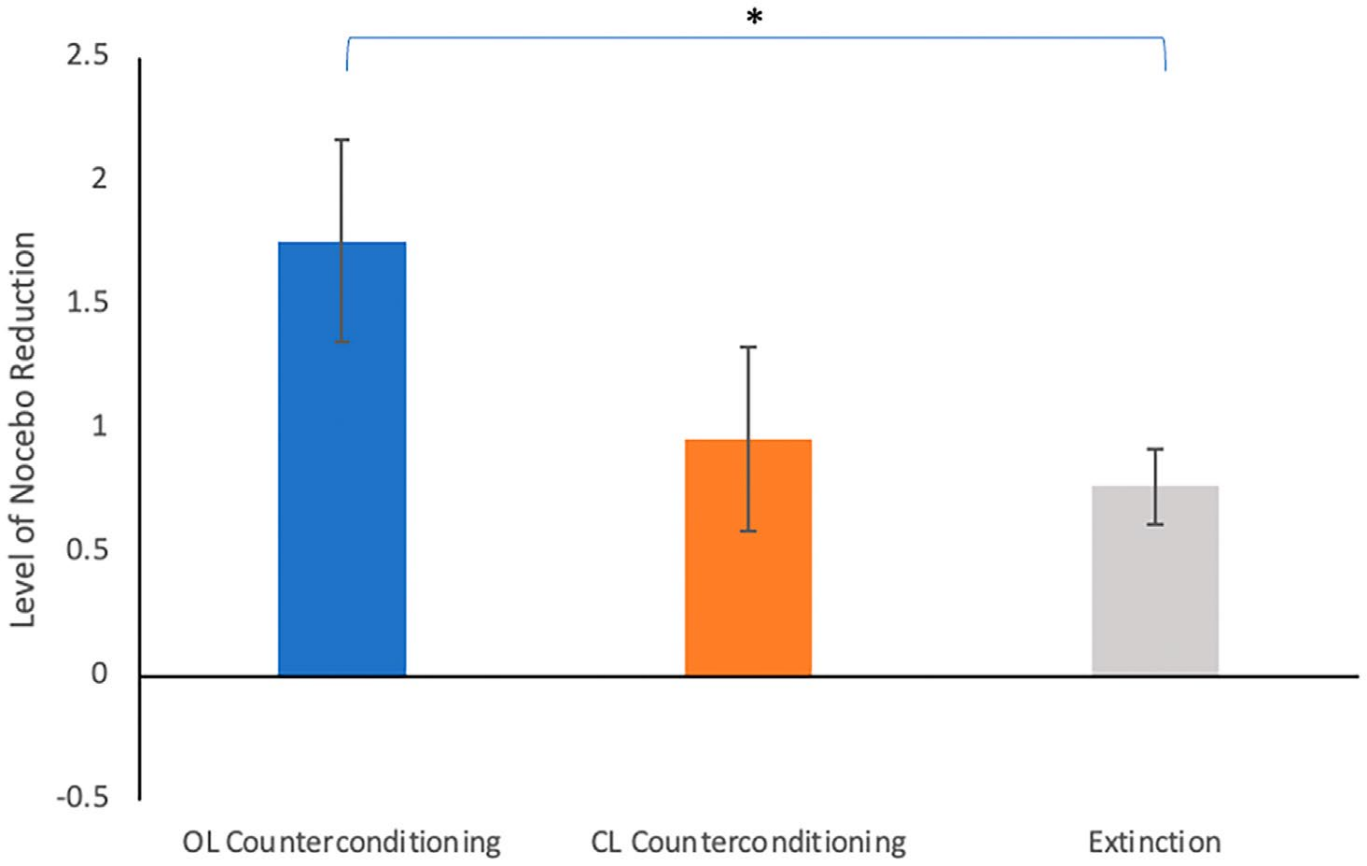
Average nocebo reduction (difference in nocebo effect after conditioning versus after counterconditioning or extinction). Open‐label counterconditioning reduced the nocebo significantly more than extinction. No other differences were found. *p < 0.01 (two‐tailed).

The nocebo effect was effectively reduced by open‐label counterconditioning (*t*(22) = 5.45, *p* < 0.001, *d* = 1.13), closed‐label counterconditioning (*t*(21) = 3.23, *p* = 0.004, *d* = 0.69), and extinction (*t*(20) = 3.60, *p* = 0.001, *d* = 0.66), which is in line with hypothesis 2. The mean reduction in each group is shown in Figure 3. Groups differed significantly in terms of nocebo reductions, as shown by a significant interaction between group and time (nocebo effect in part 1 vs. part 2), *F*(2, 63) = 3.90, *p* = 0.025, in line with hypothesis 3. A 2 × 2 mixed‐model ANOVA showed a significant interaction of open‐label counterconditioning vs. extinction with time (*F*(1, 42) = 8.00, *p* = 0.007, *d* = 0.85), which indicated open‐label counterconditioning to yield a significantly larger reduction than extinction. Two other 2 × 2 mixed‐model ANOVAs did not reveal a difference between open‐label counterconditioning and closed‐label counterconditioning (*F*(1, 43) = 2.83, *p* = 0.100, *d* = 0.50) and between closed‐label counterconditioning and extinction (*F*(1, 41) = 0.91, *p* = 0.347, *d* = 0.29) in terms of differences in nocebo reduction. These findings were partially in line with hypothesis 3, as a difference between closed‐label conditioning and extinction was also expected. To check for sensitization, pain scores on the 1st and last control trial of the learning phase were examined. Again, slight sensitization was found (on average 0.41 on the NRS).

Although no differences in strength of nocebo reduction were detected between open‐ and closed‐label counterconditioning, the groups were not found to be equivalent in efficacy, based on the 90% CI of the effect size of their difference in nocebo reduction. This falls outside of the predetermined range (90% CI [0.00, 0.20]), indicating results on either the difference or equivalency of both groups (open vs. closed‐label) are inconclusive.

**FIGURE 4:**
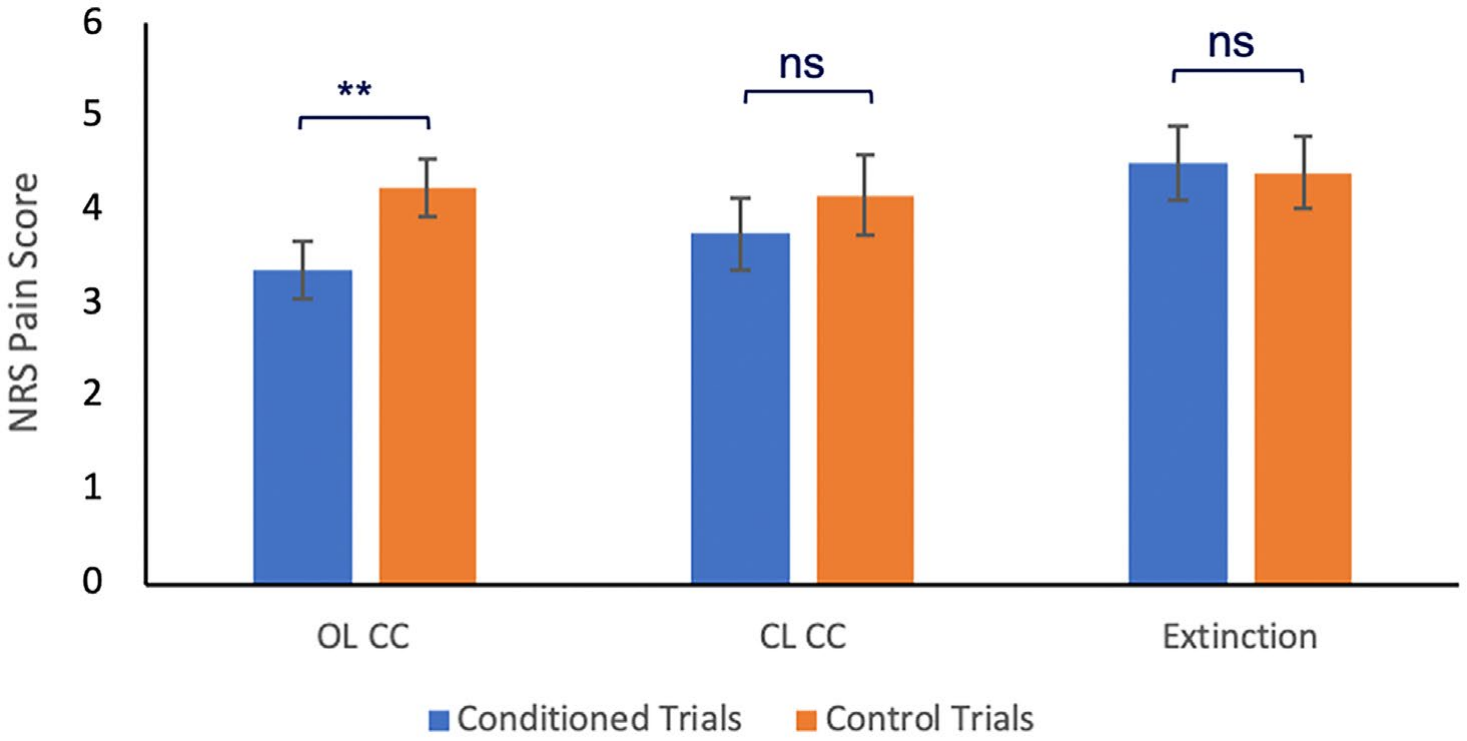
Average NRS ratings and standard error of the mean of all three experimental trials and control trials during the testing phase for both forms of counterconditioning as well as extinction. Only for open‐label counterconditioning, experimental trials were rated as significantly less painful than control trials. **p < 0.001 (two‐tailed). NRS, Numeric Rating Scale.

Finally, as can be seen in Figure 4, only in the open‐label counterconditioning group (*t*(20) = −4.20, *p* < 0.001, *d* = 0.92), experimental trials were rated as significantly less painful than control trials, implying a reversion of the nocebo effect into a placebo effect, but not in the closed‐label counterconditioning group (*t*(21) = −1.95, *p* = 0.065, *d* = 0.83) or extinction group (*t*(19) = 1.05, *p* = 0.308, *d* = 0.52). These results only partially confirm our sixth hypothesis, as a placebo effect was also expected to be found in the closed‐label group.

### 3.4 Sensitivity Analyses

After exclusion of 15 participants who did not show a nocebo effect in part 1 (i.e., 22%; 3 showed no change, 12 showed a change in the opposite direction), all previously conducted analyses of the nocebo‐reduction part were repeated. This did not yield any different results, except for the 2 × 2 mixed‐model ANOVA comparing nocebo reduction between the closed‐label counterconditioning group and extinction group, which now revealed a significant interaction between group and time (*F*(1, 33) = 6.79, *p* = 0.014, *d* = 0.45), indicating closed‐label counterconditioning to be significantly more effective in reducing the nocebo effect than extinction. Furthermore, for the closed‐label counterconditioning group, experimental trials were now rated significantly lower than control trials during the test phase of nocebo reduction (*t*(15) = −4.18, *p* < 0.001, *d* = 0.65), implying also the reversion of the nocebo effect into a placebo effect. These results would fully confirm our third and sixth hypotheses.

### 3.5 Expectations

For expected pain during nocebo induction, no differences were found between groups during the beginning of the learning phase (*F*(2, 63) = 1.261, *p* = 0.290), halfway through the learning phase (*F*(2, 63) = 0.065, *p* = 0.937), and the testing phase of nocebo induction (*F*(2, 63) = 1.158, *p* = 0.321).

Regarding expected pain during nocebo reduction, groups differed significantly on the “expected pain difference” at the beginning of the learning phase (*F*(2, 63) = 9.114, *p* < 0.001, *d* = 0.53), halfway through the learning phase (*F*(2, 63) = 14.55, *p* < 0.001, *d* = 0.69), and the testing phase of nocebo reduction (*F*(2, 62) = 22.19, *p* < 0.001, *d* = 0.95). Post hoc analyses revealed that this difference score was significantly larger (i.e., expected pain during the experimental trial was lower than during the control trial) in the open‐label counterconditioning group and closed‐label counterconditioning group compared to the extinction group, for all measurements. This was in line with the procedures, meaning the nocebo‐reduction paradigms manipulated expectations as intended.

A larger expected nocebo effect (the difference in expected pain during the first experimental and control trial) of the testing phase of nocebo conditioning was significantly related to a stronger nocebo effect (*r*(64) = 0.619, *p* < 0.001). A larger expected placebo effect in the testing phase of nocebo reduction was significantly related to a stronger nocebo reduction in the open‐label counterconditioning group (*r*(21) = −0.571, *p* = 0.007) and in the closed‐label counterconditioning group (*r*(20) = −0.718, *p* < 0.001), but not in the extinction group (*r*(19) = 0.10, *p* = 0.683). All data regarding expectations can be found in Appendix S2: Table B2.

### 3.6 Correlations of Psychological Characteristics With the Nocebo Effect and Nocebo Reduction

Correlational analyses (see Appendix S2: Table B3) showed that none of the psychological characteristics (FPQ, STAI‐T, STAI‐S, SSAS, MHLOC, EPQ) were significantly related to either the nocebo effect or its reduction.

### 3.7 Speed of Nocebo Reduction

The speed of reduction did not significantly differ between the groups, as no significant interaction between group and time was found (*F*(14.76, 464.81) = 1.51, *p* = 0.100, *d* = 0.44). The nocebo effect appeared to be reduced from the start of the reduction procedures (Appendix S2: Figure B2).

## 4 Discussion

The current experimental study investigated the efficacy of open‐label counterconditioning, closed‐label counterconditioning, and closed‐label extinction on the reduction of conditioned nocebo effects on pressure pain in healthy women. We demonstrated that open‐ and closed‐label counterconditioning (combined with verbal suggestions) and closed‐label extinction could successfully reduce nocebo effects. Open‐label counterconditioning led to a larger reduction than extinction and reversed the nocebo effect into a placebo effect, whereas extinction did not. Comparable results were found for closed‐label counterconditioning in the sensitivity analyses of nocebo responders only. Finally, lower nocebo expectancies predicted a higher response to the open‐ and closed‐label counterconditioning procedures, but not to extinction.

As for nocebo reduction, this study was the first to directly compare an open‐label counterconditioning procedure to both closed‐label counterconditioning and extinction. In line with previous studies using either open‐label (Meijer et al. 2023) or closed‐label (Bartels et al. 2017; Thomaidou et al. 2020) counterconditioning, both procedures were found to effectively reduce nocebo effects. More specifically, open‐label counterconditioning was able to fully extinguish the nocebo effect and to produce a placebo effect. Additionally, extinction led to a significant reduction of the nocebo effect, which is in line with our previous findings on open‐label extinction (Meijer et al. 2023; Thomaidou et al. 2020). Other (closed‐label) studies mentioned that nocebo effects appear to be difficult to extinguish (Bartels et al. 2017; Colagiuri et al. 2015; Colloca et al. 2008), which partially contradicts our findings. Open‐ but not closed‐label counterconditioning led to a larger reduction of the nocebo effect than extinction. However, after removing nocebo non‐responders, sensitivity analyses did show closed‐label counterconditioning to also yield a stronger reduction than extinction, in line with previous studies (Bartels et al. 2017; Meijer et al. 2023; Thomaidou et al. 2020). As for the comparison of open‐ and closed‐label counterconditioning, it was inconclusive whether both procedures were equivalent in efficacy. These results ought to be interpreted with some caution, as a larger sample size or different equivalence bounds may have yielded different results. Visual inspection of the data suggests that open‐label counterconditioning led to a somewhat larger reduction of the nocebo effect than closed‐label counterconditioning, meaning that while no statistically significant differences could be detected, the current data suggest that open‐label counterconditioning could be at least as effective or potentially even more effective than closed‐label counterconditioning. Finally, exploratory analyses revealed no differences in reduction speed for the three reduction methods, which is in line with our previous study on open‐label counterconditioning (Meijer et al. 2023). While relatively large effect sizes were reported, it cannot be ruled out that our smaller sample size may have inflated our effect sizes, just as removing non‐responders from the analyses (as shown in our sensitivity analyses). Replication of our results in larger studies may provide more insight in the true effects of our intervention.

Counterconditioning has been researched more often in the context of fear and multiple studies have found counterconditioning to successfully diminish conditioned effects (Gatzounis et al. 2021; Kang et al. 2018; Meulders et al. 2015; Raes and De Raedt 2012). Results on counterconditioning being more effective than extinction are mixed in fear research (Jozefowiez et al. 2020). However, counterconditioning appears to be a promising strategy for reducing nocebo effects (Bartels et al. 2017; Meijer et al. 2023), and based on our results, (open‐label) counterconditioning appears to be more effective than extinction. Consequently, counterconditioning‐based interventions may be more beneficial than treatments based on extinction (such as exposure therapy). For example, the procedures used in the current study could be translated to a more clinical setting, using a treatment protocol containing multiple sessions, in order to reduce nocebo effects on (chronic) pain (Meijer et al. 2022). By repeating the counterconditioning procedure over multiple sessions and adding homework exercises, experimental results from the lab may be translated to clinical pain symptoms. Additionally, when nocebo effects occur in a clinical situation (e.g., patients experiencing nausea upon entering the hospital), it may be helpful to educate patients on the nocebo effect and explain how their symptom was conditioned.

Before these procedures can be applied in clinical practice, however, there is a need for replication in clinical samples and the intervention will have to be translated to real‐world settings. This may be challenging, as it is hard to precisely determine how potential nocebo effects in patients have been induced and whether and how conditioning played a role in nocebo induction. Furthermore, in a healthy sample who are only experiencing acute pain during the experiment, it may be easier to influence expectations than in a clinical sample with an extensive background of negative experiences with pain and/or treatment.

Future studies could look further into the possible application of the proposed procedures and investigate which elements of the intervention could be used and tailored to patients in clinical settings. For example, placebo‐conditioning procedures could be used on top of a treatment to create positive associations with this treatment. While not formally counterconditioning, procedures similar to our current paradigm may prove beneficial. It should also be explored whether such interventions are feasible to use in patients already undergoing (several) treatments, as this may add more burden. Ideally, (elements of) the intervention could be combined with existing treatments and/or patients could be educated on the occurrence of nocebo effects and how conditioning may play a part in this.

In our study, expectations were significantly correlated to nocebo induction and reduction in the counterconditioning groups. A previous study in a clinical sample has shown that open‐label placebos have a beneficial effect that is unrelated to positive expectancies induced by positive suggestions about the placebo, but the underlying mechanism of the open‐label placebo effect remained unclear (Schaefer et al. 2018b). Other research on deceptive placebo and nocebo effects has shown expectations to be related to these effects, and while in open‐label paradigms people are told the treatment is a sham, positive suggestions are typically still provided, suggesting a beneficial effect regardless of the nature of the treatment, which is in line with our results (Kelley et al. 2012; Sandler and Bodfish 2008). Our current study included only healthy participants, in which expectancy effects may have been stronger than in a clinical sample. Especially for patients with repeated negative experiences, it may be harder to have positive expectations regarding an intervention. Resultantly, in clinical samples expectations may play a smaller role in explaining open‐label placebo effects (Kaptchuk 2018). Future studies could look further into the underlying mechanisms of open‐label counterconditioning, comparing healthy and patient groups, as to target the relevant mechanisms specifically when designing interventions.

A limitation of the study might be a possible response bias of open‐label conditions. Particularly, the open‐label suggestions leave less uncertainty about the pain that participants are about to receive, which may be supported by the fact that participants in the closed‐label group expected slightly more pain during experimental trials than in the open‐label group at the start of the learning phase of nocebo reduction. Additionally, participants in the open‐label group may be more prone to a response bias. No significant response bias was found based on the exit questionnaire, but as this conclusion is based on a single self‐report question, this result may not be fully reliable. It therefore cannot be ruled out that participants in the open‐label group changed their answers unconsciously, merely as there was no uncertainty regarding the difference in intensity between experimental and control trials (in contrast to the closed‐label group). Future research could look into adding other measures of pain that are not sensitive to response bias, such as skin conductance or heart rate variability, although these measures do not measure pain directly (Forte et al. 2022; Hu et al. 2021). Furthermore, non‐verbal pain indicators such as facial grimaces or bracing could be explored and added to the experiment (Feldt 2000).

Furthermore, the difference in nocebo reduction between both counterconditioning groups and the extinction groups may reflect a difference in induced expectations caused by the suggestions provided in the counterconditioning groups. In the current set‐up, it cannot be determined whether superiority of counterconditioning over extinction is a result of the suggestions provided in the counterconditioning groups or of the counterconditioning procedure itself. Nevertheless, extinction (without the provision of explicit verbal suggestions) does appear to best mimic a natural history group, in which no specific interventions are performed. While relevant to investigate whether the suggestions, the counterconditioning procedure, or the combination of both contributed most to nocebo reduction, the main goal of the current study was to explore whether a group receiving such a counterconditioning intervention would benefit from more nocebo reduction than a group in which no active intervention was provided (i.e., extinction). Future research could look into the separate and combined effects of the two manipulations within the counterconditioning procedure, in order to determine the underlying mechanism(s). While the use of pressure pain is highly relevant for people with musculoskeletal pain, another limitation of the study is that the device used administered pressure repeatedly to the thumbnail of the nondominant hand, which could lead to sensitization or habituation. Indeed, in the current study slight sensitization was seen from the first learning trial to the last learning trial of nocebo conditioning, with somewhat higher sensitization for the experimental than control trials. This indicates that participants sensitised more on the trials using higher pressure intensities, meaning the difference between experimental and control trials became larger throughout the experiment. However, sensitization was similar in all three groups, meaning it likely did not influence results on efficacy of the reduction methods. In our current set‐up, switching sites for pain administration was not possible. In future studies however, it could be worthwhile to repeatedly change the location to which the pressure is administered (similarly to studies using heat or electrical pain), as this may lead to less sensitization or habituation.

Next, only female participants were tested in our study. While fibromyalgia is more prevalent in women, gender differences in the induction and reduction of nocebo effects could have influenced our results and limited the generalizability of our findings to other genders, as well as to other (pain) syndromes. Reviews on sex differences in placebo and nocebo effects have concluded that conditioning, as compared to verbal suggestions, induces stronger responses in females, while verbal suggestions, as compared to conditioning, create stronger responses in males. Furthermore, females respond more strongly to nocebo treatment, whereas males responded stronger to placebo treatment (Enck and Klosterhalfen 2019; Vambheim and Flaten 2017). In our study, we used both suggestions and conditioning, meaning no gender differences may have been found, except for differences in sensitivity to placebo versus nocebo effects. Nevertheless, it would be relevant to also investigate potential gender differences in our (experimental) paradigm, as to be able to tailor future interventions to the individual patient.

In conclusion, this study demonstrates that conditioned nocebo effects on pressure pain can successfully be reduced by both open‐ and closed‐label counterconditioning, as well as extinction, with the largest nocebo reduction being found after open‐label counterconditioning. While more research is needed on the potential superiority of counterconditioning over extinction in both healthy and clinical samples, and the mechanisms responsible for the open‐ and closed‐label counterconditioning effects, the current study demonstrates that open‐label counterconditioning may be a promising new strategy for reducing nocebo effects in a non‐deceptive and ethical manner, which may be used in a variety of patient populations, such as patients with chronic pain.

## Author Contributions

This study was designed by S.M., H.M., K.J.P., and A.W.M.E. The experiments were performed by S.M. The data were analysed by S.M., and the results were critically examined by all authors. S.M. had a primary role in preparing the manuscript, which was edited by H.M., K.J.P., and A.W.M.E. All authors have approved the final version of the manuscript and agree to be accountable for all aspects of the work.

## Funding

This study was funded by a VICI grant from the Netherlands Organization for Scientific Research (NWO; grant number 45316004) and an NWO Stevin grant, awarded to A. W. M. Evers.

## Conflicts of Interest

The authors declare no conflicts of interest.

## Supporting information


**Appendix S1:** ejp70248‐sup‐0001‐AppendixS1.pdf.

**Appendix S2:** ejp70248‐sup‐0002‐AppendixS2.docx.
