# Supplementary material for: Can Nocebo Effects Be Reduced via Open‐ and Closed‐Label Counterconditioning?
Source: Eur J Pain. 2026 Mar 12;30(3):e70248. doi: 10.1002/ejp.70248 (PMC12982913; doi:10.1002/ejp.70248)

DERMAL NERVE STIMULATION (DNS)

WAT IS DNS?

Een Dermal Nerve Stimulation (DNS) apparaat is een apparaat dat zenuwen stimuleert via de toediening van elektrische signalen. Deze stimulatie zorgt ervoor dat communicatie tussen zenuwcellen sneller verloopt.

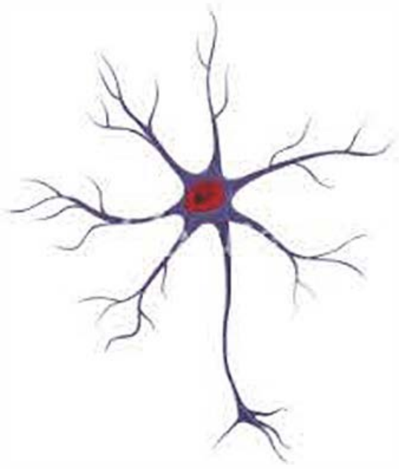

HOE WERKT DNS?

Zenuwen in de huid communiceren door signalen naar het ruggenmerg te sturen via elektrische signalen. Het DNS-apparaat kan deze signalen beïnvloeden waardoor de intensiteit van binnenkomende prikkels, zoals pijn, wordt verhoogd. Wanneer deze signalen verzonden worden naar het ruggenmerg en vervolgens het brein, wordt men bewust van de pijnsensatie.

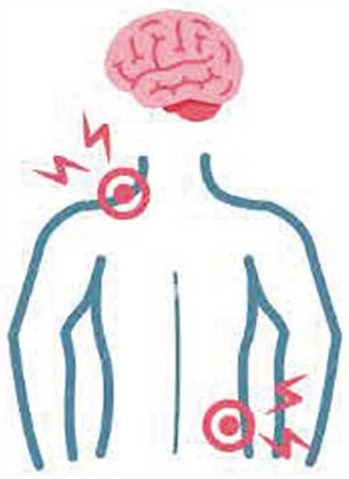

HOE WORDT DNS TOEGEPAST?

Het DNS-apparaat dient elektrische signalen toe via elektroden op de huid. Een voordeel van DNS is dat deze lichte, (bijna) niet merkbare, signalen voldoende zijn om de communicatie tussen zenuwcellen te beïnvloeden en daarmee pijnsensaties te veranderen.

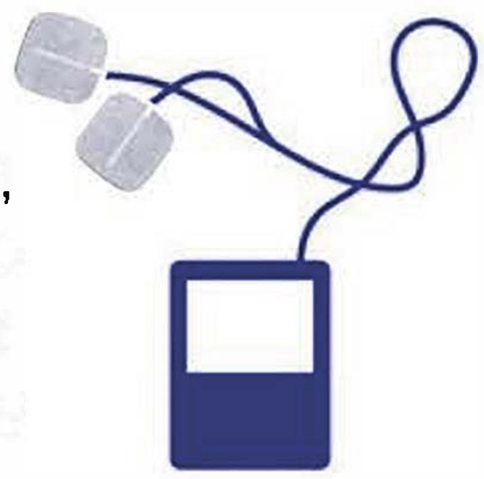

Supplement: Supplementary file 1 — Appendix S1: ejp70248‐sup‐0001‐AppendixS1.pdf. [file EJP-30-0-s002.pdf]
