# Supplementary material for: Can Nocebo Effects Be Reduced via Open‐ and Closed‐Label Counterconditioning?
Source: Eur J Pain. 2026 Mar 12;30(3):e70248. doi: 10.1002/ejp.70248 (PMC12982913; doi:10.1002/ejp.70248)
Supplement: Supplementary file 2 — Appendix S2: ejp70248‐sup‐0002‐AppendixS2.docx. [file EJP-30-0-s001.docx]

**Appendix B – Supplementary tables and figures**

**Table B1***Group means and SDs for calibration values (preceding group randomization) and exit questions*

|  | **All participants** | |  | | | **Subgroups** | |  |  |
| --- | --- | --- | --- | --- | --- | --- | --- | --- | --- |
|  | **Nocebo conditioning (n=66)** | | **Open-label counterconditioning (n=23)** | | | **Closed-label  counterconditioning (n=22)** | | **Extinction (n=21)** | |
|  | Mean | SD | Mean | | SD | Mean | SD | Mean | SD |
| **Calibrations** |  |  |  |  | |  |  |  |  |
| No pain (kg/cm^2^) | 2.53 | 1.18 | 2.59 | 1.25 | | 2.49 | 1.47 | 2.50 | 0.60 |
| Slight pain (kg/cm^2^) | 4.62 | 1.52 | 4.43 | 1.67 | | 4.84 | 1.75 | 4.55 | 1.02 |
| Moderate pain (kg/cm^2^) | 7.27 | 2.38 | 6.72 | 2.50 | | 7.57 | 2.73 | 7.60 | 2.73 |
| **Exit Questionnaire** |  |  |  |  | |  |  |  |  |
| Focus during experiment | 6.75 | 2.32 | 6.68 | 2.05 | | 7.10 | 2.33 | 6.46 | 2.63 |
| Trust in experimenter | 9.08 | 1.14 | 8.94 | 1.29 | | 9.29 | 0.83 | 9.00 | 1.24 |
| Competence experimenter | 9.30 | 1.13 | 9.15 | 1.49 | | 9.42 | 0.76 | 9.32 | 1.02 |
| Response bias | 0.51 | 0.94 | 0.74 | 1.19 | | 0.39 | 0.72 | 0.36 | 0.79 |


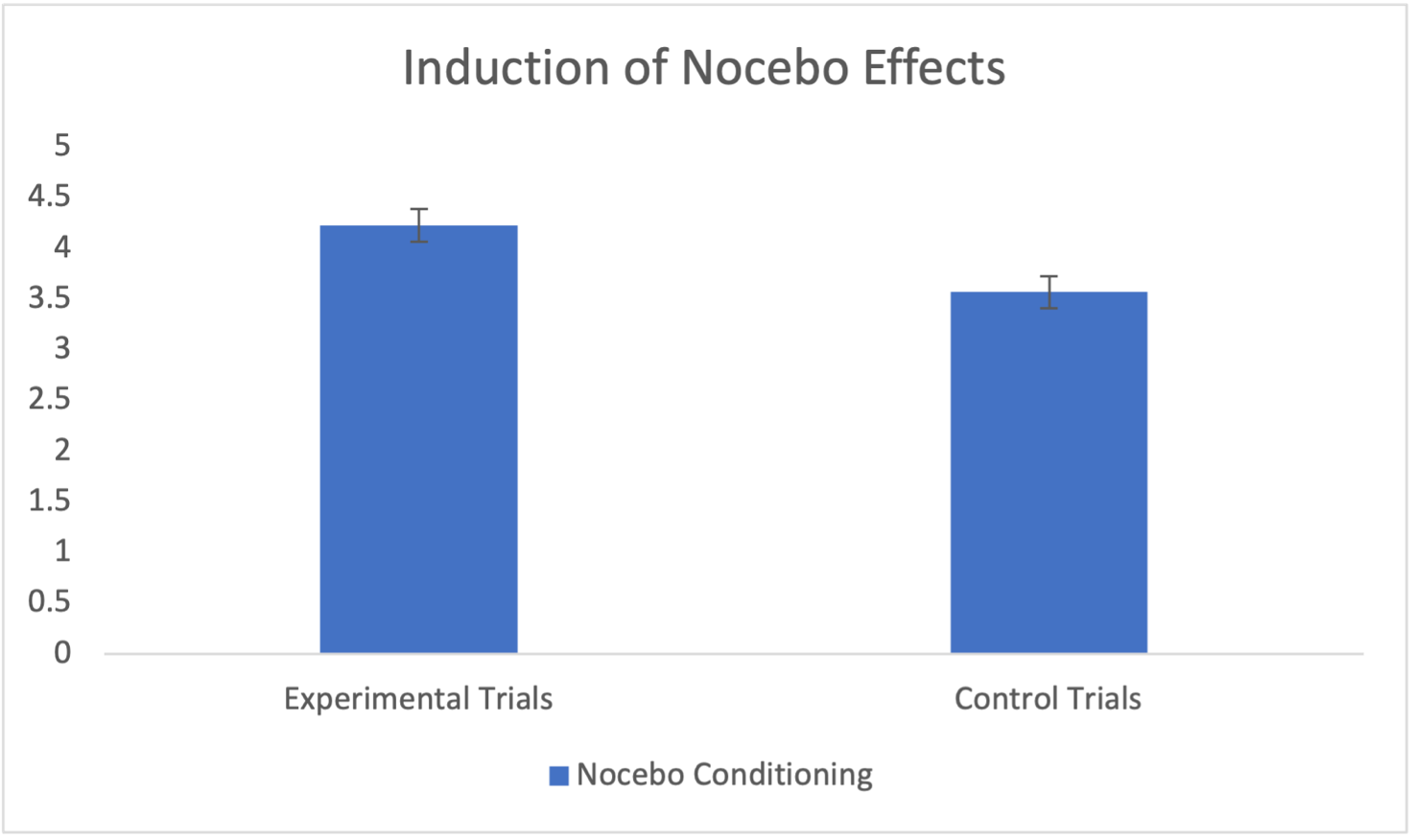


**Fig B1** . Average pain ratings (NRS) and Standard Error of the Mean (SEM) of all 3 experimental trials and control trials during the testing phase of nocebo conditioning. ** *p* < .001 (two-tailed).

**Table B2**
*Group means and standard deviations for expected pain during the 1^st^ and 10^th^ experimental and control trial during the learning phase of nocebo induction and reduction, as well as expected pain during the 1^st^ experimental and control trial of the testing phase of both procedures.*

|  | **All participants**  **(n=66)** | | **Open-label counter-conditioning**  **(n=23)** | | **Closed-label counter-conditioning**  **(n=22)** | | **Extinction**  **(n=21)** | |
| --- | --- | --- | --- | --- | --- | --- | --- | --- |
|  | Mean | SD | Mean | SD | Mean | SD | Mean | SD |
| **Pain expectancies (0-10 NRS)**  **Nocebo induction** |  |  |  |  |  |  |  |  |
| 1^st^ experimental trial learning  phase | 3.61 | 1.75 | 3.88 | 1.66 | 3.30 | 1.30 | 3.65 | 2.23 |
| 1^st^ control trial learning  phase | 3.14 | 1.42 | 3.67 | 1.55 | 2.97 | 0.97 | 2.74 | 1.53 |
| 10^th^ experimental trial learning  phase | 5.09 | 1.40 | 5.10 | 1.36 | 5.05 | 1.45 | 5.10 | 1.46 |
| 10^th^ control trial learning  phase | 3.68 | 1.42 | 3.64 | 1.29 | 3.74 | 1.44 | 3.67 | 1.59 |
| 1^st^ experimental trial testing  phase | 5.70 | 1.30 | 5.67 | 1.22 | 5.61 | 1.30 | 5.83 | 1.43 |
| 1^st^ control trial testing  phase | 3.70 | 1.33 | 3.55 | 1.26 | 4.00 | 1.25 | 3.56 | 1.49 |
| **Nocebo reduction** |  |  |  |  |  |  |  |  |
| 1^st^ experimental trial learning  phase |  |  | 3.37 | 1.59 | 4.05 | 1.49 | 5.86 | 1.91 |
| 1^st^ control trial learning  phase |  |  | 3.56 | 1.27 | 3.47 | 1.29 | 3.19 | 1.25 |
| 10^th^ experimental trial learning  phase |  |  | 2.46 | 1.22 | 3.25 | 1.62 | 4.64 | 1.67 |
| 10^th^ control trial learning  phase |  |  | 4.30 | 1.34 | 4.13 | 1.62 | 3.75 | 1.18 |
| 1^st^ experimental trial testing  phase |  |  | 2.15 | 1.10 | 2.47 | 1.65 | 4.80 | 1.89 |
| 1^st^ control trial testing  phase |  |  | 4.23 | 1.55 | 4.20 | 1.85 | 3.84 | 1.87 |

**Table B3**Pearson correlations of sum scores on the personality questionnaires with the strenght of the nocebo effect (all participants) and amount of nocebo reduction in the different subgroups. No significant correlations were found after correction for multiple testing (*p* < .0011).

|  | Nocebo effect | Nocebo reduction (OL-CC) | Nocebo reduction (CL-CC) | Nocebo reduction (extinction) |
| --- | --- | --- | --- | --- |
| Fear of Pain^1^ | -.038 | -.046 | -.044 | .034 |
| State anxiety^2^ | -.037 | -.051 | -.153 | .221 |
| Trait anxiety^3^ | -.172 | -.229 | .015 | -.009 |
| Internal Locus of Control^4^ | .152 | .312 | -.067 | .042 |
| Locus of control -Powerful others^5^ | .171 | .545 | -.029 | .011 |
| Locus of control -Chance^6^ | .141 | .330 | .185 | -.135 |
| Somatosensory amplification^7^ | -.105 | -.213 | -.267 | .193 |
| Psychoticism^8^ | -.094 | .014 | .326 | -.376 |
| Neuroticism^8^ | -.133 | -.310 | -.381 | .153 |
| Extraversion^8^ | .194 | .328 | .233 | .061 |
| Social desirability^8^ | .310 | .479 | -.052 | -.015 |

*1. Fear of Pain Questionnaire, 2. State-Trait Anxiety Inventory, State Scale Short form, 3. State-Trait Anxiety Inventory, Trait Scale, 4. Multidimensional Health Related Locus of Control (scale for internal locus of control), 5. Multidimensional Health Related Locus of Control (scale for* *external locus of control; powerful others), 6. Multidimensional Health Related Locus of Control (scale for* *external locus of control; chance), 7. Somatosensory Amplification Scale, 8. Eysenck Personality Questionnaire (Revised Short Scale)*


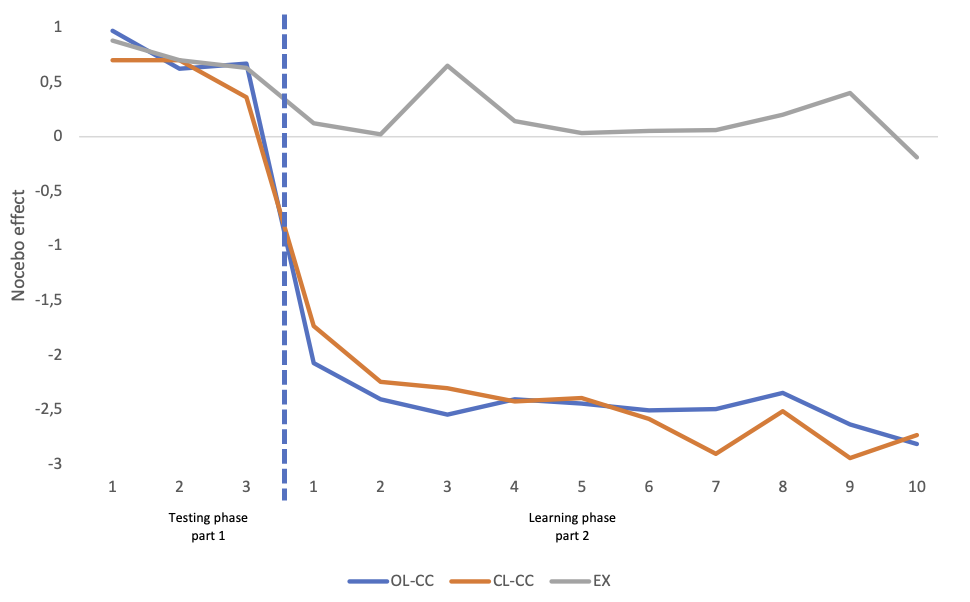
**Fig. B2.** The nocebo effect throughout the experiment are displayed for open-label counterconditioning (OL-CC), closed-label counterconditioning (CL-CC) and extinction (EX). The first 3 trials represent the testing phase of part 1, while the next 10 trials represent the learning phase of part 2 (separated by the vertical line). While both counterconditioning groups show the largest reduction, the speed of reduction does not significantly differ between the groups, as no significant interaction between group and time was found (F(14.76, 464.81) = 1.51*, p* = .100, *d* = .44).
